# Supplementary material for: Causal interplay between lactose intolerance and gut microbiota: a combined bidirectional Mendelian randomization and in vivo validation study
Source: Front Nutr. 2026 Jun 1;13:1803337. doi: 10.3389/fnut.2026.1803337 (PMC13265576; doi:10.3389/fnut.2026.1803337)
Supplement: Supplementary file 1 [file Data_Sheet_1.zip › supplementary materials/Forward/leave one out cross validation/ebi-a-GCST90027591.finngen_R12_E4_LACTONAS.pdf]

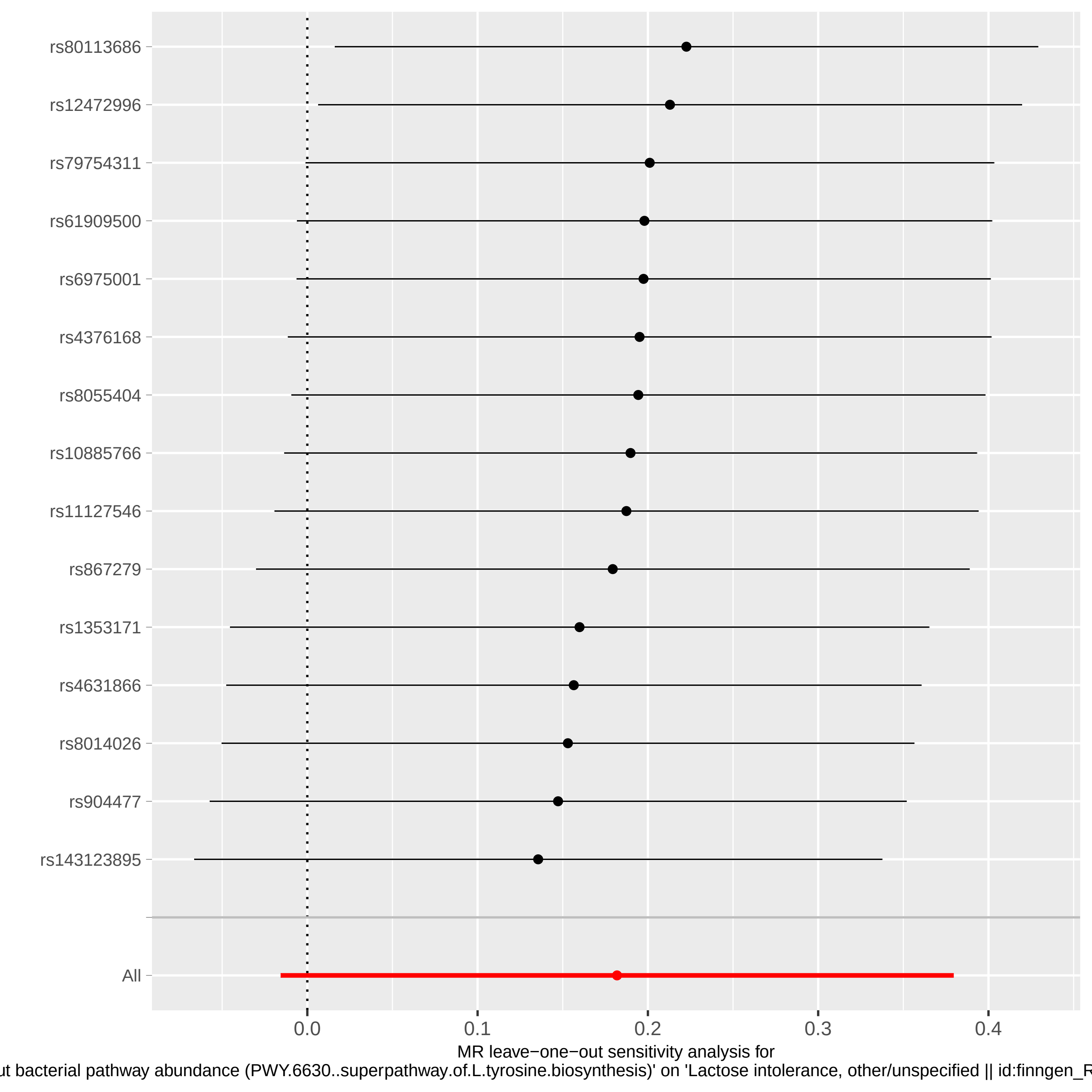
rs80113686
rs12472996
rs79754311
rs61909500
rs6975001
rs4376168
rs8055404
rs10885766
rs11127546
rs867279
rs1353171
rs4631866
rs8014026
rs904477
rs143123895
All
0.0
0.1
0.2
0.3
0.4
MR leave−one−out sensitivity analysis for
ut bacterial pathway abundance (PWY.6630..superpathway.of.L.tyrosine.biosynthesis)' on 'Lactose intolerance, other/unspecified || id:finngen_R
